# Case Report: The potential association with polyglandular autoimmune syndrome in a dog following long-term oclacitinib therapy

**DOI:** 10.3389/fvets.2025.1535272

**Published:** 2025-02-27

**Authors:** Kwang-Sup Lee, Jun-Won Yoon, Tae-Jung Dan, Sang-Min Lim, Hee-Jung Jeon, Mi-Ae Kang, Chan-Sik Nam, Hee-Myung Park

**Affiliations:** ^1^Department of Veterinary Internal Medicine, College of Veterinary Medicine, Konkuk University, Seoul, Republic of Korea; ^2^IBYU Animal Hospital, Seo-gu, Incheon, Republic of Korea; ^3^VET and GENE, Jungwon-gu, Gyeonggi-do, Republic of Korea

**Keywords:** polyglandular autoimmune syndrome, 21-hydroxylase autoantibodies, thyroglobulin autoantibodies, IL-10, oclacitinib

## Abstract

A 12-year-old spayed female Maltese dog had been receiving oclacitinib, a Janus kinase (JAK) inhibitor, for 7 years to manage chronic pruritus due to atopic dermatitis. During this treatment, the dog was diagnosed with primary hypoadrenocorticism and hypothyroidism based on history, physical examination, and hormonal analysis. This case was initially suspected to be polyglandular autoimmune syndrome (PAS) based on long-term treatment of oclacitinib. To confirm the diagnosis of PAS, the presence of autoantibodies was tested. 21-hydroxylase autoantibodies (21-OHAb) were detected, but negative for thyroglobulin autoantibodies (TgAA). Considering the potential of oclacitinib to induce autoimmune diseases, we examined to identify the association of interleukin-10 (IL-10) in PAS of the dog. This case suggests a potential association between prolonged oclacitinib administration and the development of PAS in a dog. Regular hormonal monitoring and careful dose adjustments of oclacitinib during long-term therapy of atopic dermatitis are recommended to minimize the risk of autoimmune disease development. To the best of our knowledge, this is the first case report suggesting that PAS could be induced by oclacitinib.

## Introduction

Polyglandular autoimmune syndrome (PAS) in dogs is a multifaceted disorder characterized by the simultaneous dysfunction of multiple endocrine organs due to autoimmune processes. The etiology of PAS is believed to be multifactorial, involving both genetic predispositions and environmental triggers (1). PAS in dogs is a rare but increasingly recognized condition characterized by autoimmune dysfunction affecting multiple endocrine organs (2, 3). Although infrequently documented, the growing number of cases underscores the clinical importance of considering PAS in the differential diagnosis, particularly in dogs presenting with concurrent endocrine abnormalities. This study contributes to the limited veterinary literature by exploring a potential association between PAS and the administration of oclacitinib, an immunomodulatory drug commonly prescribed for atopic dermatitis. The pathogenesis of PAS in dogs remains poorly understood; however, several hypotheses have been proposed. Genetic predisposition likely plays a central role, as suggested by parallels with human PAS, where specific HLA (Human Leukocyte Antigen) haplotypes are strongly associated with autoimmune diseases (4–6). Environmental factors, such as medications that modulate immune responses, may act as critical triggers, potentially unmasking latent autoimmune tendencies in genetically predisposed individuals (7, 8).

To date, six case reports of canine PAS have been documented. This includes two reports from 2021 describing cases involving both diabetes mellitus and adrenal insufficiency (9, 10), as well as additional reports published between 1995 and 2021 that highlight concurrent hypothyroidism and adrenal insufficiency (11–15). Despite the growing recognition of PAS in veterinary literature, the underlying mechanisms and potential environmental triggers remain poorly understood.

In recent years, the focus has shifted toward exploring both genetic predispositions and environmental factors that may contribute to the onset of autoimmune disorders, including PAS. One such environmental factor is the administration of immunomodulatory drugs. To the best of our knowledge, this study is the first to investigate the potential association between the administration of Oclacitinib (Apoquel^®^, Zoetis, USA), an immunomodulatory drug commonly used to treat atopic dermatitis, and the onset of PAS in a dog.

## Case description

A 12-year-old spayed female Maltese had been receiving oclacitinib for the treatment of atopic dermatitis for 7 years. No remarkable findings were noted on physical examination, except for lethargy and abdominal distension, which led to the discontinuation of the medication. Consequently, complete blood count, serum chemistry, and electrolyte tests were conducted.

In the complete blood count, anemia was observed [red blood cell (RBC): 4.12 × 10^12^/L; reference range: 5.5–8.5 × 10^12^/L; hematocrit (HCT): 31.8%; reference range: 39–56%] without regeneration. Additionally, there were elevated platelet counts [491 K/μL; reference range: 117–460 K/μL]. Serum biochemistry tests revealed elevated alanine aminotransferase (ALT) [106 U/L; reference range: 0–88 U/L], alkaline phosphatase (ALKP) [220 U/L; reference range: 0–212 U/L], bile acids [54.2 μmol/L; reference range: 0–30 μmol/L], and total cholesterol (T-Chol) [462 mg/dL; reference range: 111–312 mg/dL]. Electrolyte testing showed hyperkalemia [6.3 mmol/L; reference range: 3.5–5.8 mmol/L], with an Na/K ratio of 24.4 [reference range: >27] (Table 1). Based on the clinical signs, hematology and biochemical profiles, we suspected hypoadrenocorticism and an adrenocorticotropic hormone (ACTH) stimulation test was initially performed to rule out a hormonal disorder.

**Table 1:** Results of a complete blood count (CBC), serum chemistry, and electrolyte.

**Parameters**	**Results**	**Reference range**
**Complete blood count (CBC)**		
RBC	4.12	5.5–8.5 × 10^12^/L
HCT (%)	31.8	39–56
PLT	491	117–460 K/μL
**Chemistry**		
ALT	106	0–88 U/L
ALKP	220	0–212 U/L
Bile acids	54.2	0–30 μmol/L
T-CHOL	462	111–312 mg/dL
**Electrolytes**		
Na^+^	154	138–160 mmol/L
K^+^	6.3	3.5–5.8 mmol/L
Na/K Ratio	24.4	>27

RBC, red blood cell count; HCT, hematocrit; PLT, platelets; ALT, alanine aminotransferase; ALKP, alkaline phosphatase; T-CHOL, total cholesterol; Na^+^, sodium; K^+^, potassium; Na/K, sodium-to-potassium ratio.

The ACTH stimulation test results were as follows: basal cortisol level < 0.001 μg/dL (reference range 2–6 μg/dL) and post-ACTH cortisol level 0.1 μg/dL (reference range 6–18 μg/dL; Table 2), which is consistent with a diagnosis of Addison's disease. The dog was treated with prednisolone (Solondo^®^, 0.2 mg/kg, PO, SID; Yuhan Corporation, South Korea) and desoxycorticosterone pivalate (Zycortal^®^, 2.2 mg/kg, SC, every 28 days; Dechra Veterinary Products, United Kingdom). After 2 months of monitoring, symptoms were observed, including bruises without trauma, alopecia on the bridge of the nose, tail alopecia, general alopecia, excessive sleeping and lethargy, and sensitivity to cold. Consequently, a thyroid panel was examined at Green Vet Diagnostic Laboratory (Yongin-si, South Korea). The results showed that the total thyroxine (T4) level was < 0.5 μg/dL (reference range: 1.1–4.6 μg/dL), the free T4 level was < 0.3 μg/dL (reference range: 0.6–3.7 μg/dL), and the canine thyroid stimulating hormone (TSH) level was 0.69 ng/mL (reference range: 0.05–0.42 ng/mL; Table 2). Based on these results, hypothyroidism was additionally diagnosed, and the dog was treated with levothyroxine sodium oral solution (Leventa^®^, 0.01 mg/kg, PO, SID; MSD Animal Health, United Kingdom). Due to the presence of bruises without trauma, acquired von Willebrand syndrome secondary to primary hypothyroidism was suspected, and vWF levels were measured using an ELISA (Animal Health Diagnostic Center, Cornell University, Ithaca, NY, USA), indicated that a vWF level of 30% (reference range, 70–180%) was into the “carrier range” (< 50%), suggesting an elevated risk of abnormal bleeding and potential transmission of vWD.

**Table 2:** Results of adrenal gland and thyroid hormonal tests.

**Test**	**Results**	**Reference range**
ACTH stimulation (Pre)	< 0.001	2 ~ 6
ACTH stimulation (Post)	0.1	6 ~ 18
T4, Total	< 0.5	1.1 ~ 4.6 (ug/dL)
Free T4	< 0.3	0.6 ~ 3.7 (ug/dL)
Canine TSH	0.69	0.05 ~ 0.42 (ng/mL)

ACTH, adrenocorticotrophic hormone; T4, Thyroxine; TSH, thyroid-stimulating hormone.

Given the concurrent occurrence of hypoadrenocorticism and hypothyroidism, PAS was suspected. To confirm autoimmune disorders, we measured 21-OHAb and TgAA (Table 3). For 21-OHAb quantification, an ELISA was conducted using the Canine Anti-Cytochrome P450c21/21-Hydroxylase (CYP21B) Antibody ELISA Kit (MyBioSource, San Diego, CA, USA) according to the manufacturer's instructions. This assay was conducted as described previously (16) utilizing 21-OH as the antigen to detect anti-adrenal autoantibodies. An increased serum concentration of 21-OHAb was confirmed by optical density (OD) values of 1.475 and 1.465, measured with serum samples collected twice at a 1-month interval, exceeding the positive threshold of 0.2 and suggesting immune-mediated adrenalitis (16). To evaluate thyroid autoimmunity, TgAA levels were measured at IDEXX Reference Laboratories (Westbrook, ME, USA). The 4% results of TgAA (the negative range, < 20%) in a repeated test 1 month later was considere to be negative.

**Table 3:** Results of autoantibody test against adrenal gland and thyroid gland.

**Test**	**Sample 1 (2024-05-01)**	**Sample 2 (2024-06-05)**	**Reference range**
21-OHAb (OD)	1.475	1.465	Hypoadrenocorticism > 0.2
TGAA Screen (%)	4	4	Negative < 20%

21-OHAb, 21-hydroxylase autoantibody; TgAA, thyroglobulin autoantibody; OD, optical density.

As an additional diagnostic measurement, serum interleukin-10 (IL-10) levels were measured to identify its potential role in immune regulation, as decreased IL-10 has been associated with the development of autoimmune diseases. The IL-10 concentration in the affected dog was 91.66 pg/mL. In comparison, the mean IL-10 concentration in healthy controls (*n* = 9) was 88.55 ± 11.75 pg/mL (standard error of the mean, SE), with a 95% confidence interval (CI) of 65.52–111.58 pg/mL.

At the follow-up visit 28 days later, the dog showed improved overall condition, with resolved bruising, increased activity, and no remarkable clinical signs. The owner adhered to the prescribed treatment without any difficulties, and the dog tolerated the medications well, experiencing no adverse or unanticipated events. However, additional diagnostic tests were not performed due to the owner's financial constraints.

## Discussion

In this case, there was a decrease in RBC and HCT levels. The causes of this decrease can be attributed to hormonal influences. Steroid hormones and thyroid hormones play a crucial role in hematopoiesis, and the patient's adrenal insufficiency and hypothyroidism likely contributed to the reduced concentrations of cortisol and thyroid hormones observed. The deficiency of steroid hormones leads to a decrease in HCT and RBC counts. Additionally, hypothyroidism suppresses bone marrow activity, reducing erythropoiesis, which can result in non-regenerative anemia. Thyroid hormones stimulate the proliferation of erythroid progenitor cells either directly or by increasing the production of erythropoietin. Second, bleeding may have occurred, further reducing HCT and RBC levels. The observed bruising in this case reflects acquired von Willebrand syndrome due to hypothyroidism, which impairs the synthesis or secretion of vWF, leading to an increased tendency for bruising (17). Thus, hypothyroidism can induce an increase in platelet count, but a decrease in platelet function.

Abnormalities of electrolyte profiles in this case were attributed by both aldosterone and cortisol deficiencies of typical Addison's disease. However, we examined the presence of 21-OHAb and, if it is present, it can suggest that this adrenal insufficiency can be associated with autoimmune etiology. Subsequently, this indicates the destruction of both the zona glomerulosa (ZG), responsible for aldosterone production, and the zona fasciculata (ZF), responsible for cortisol production, in the adrenal cortex with immune-mediated process. Thus, the presence of 21-OHAb in serum can support a diagnosis of autoimmune-mediated Addison's disease.

In the serum chemistry analysis, a slight elevation in liver parameters, including ALT, ALP, bile acids, and cholesterol levels, is observed. These findings may be indicative of hypothyroidism. Hypothyroidism in dogs can lead to increased liver enzyme levels due to the slowed metabolic rate that affects various bodily functions. The thyroid hormones are crucial for regulating metabolism, and when they are deficient, it can result in an accumulation of substances like triglycerides and cholesterol in the blood, which in turn can affect liver function. Also, hypothyroidism can lead to weight gain and obesity, which are also associated with elevated liver enzymes. Additionally, during the thyroid panel test, TSH levels were elevated, which excludes the possibility of euthyroid sick syndrome. The distinction is made based on whether TSH is normal or elevated; if TSH is elevated, it is typically considered primary hypothyroidism. In this case, while it is possible that prior administration of prednisone (PDS) could secondarily decrease T4 levels before the diagnosis of hypothyroidism, the presence of elevated TSH leads to the conclusion that the hypothyroidism was not secondary to PDS treatment for Addison's disease.

Despite the detection of hypothyroidism, TgAA tests were negative in this case. It appears that lymphocytic thyroiditis progresses through four distinct stages. Initially, there is local lymphocytic infiltration along with the presence of positive autoantibodies. As the disease advances, more than 60 to 70% of the thyroid gland is destroyed, leading to a compensatory increase in TSH to maintain normal T4 levels. Over the course of 1 to 3 years, this destructive process causes the gradual onset of clinical signs, with a decline in serum thyroid hormone levels and a further rise in TSH. As the destruction continues, most of the functional thyroid tissue is lost. In the final stage, the thyroid is replaced by fibrous and adipose tissue, with the disappearance of inflammatory cells and circulating autoantibodies (18, 19). In this case, given that the dog has been on oclacitinib for 7 years, it is likely that enough time has passed for the disease to have progressed to the final stage. Moreover, the diagnostic value of TgAA as a biomarker for hypothyroidism has not been definitively established (18). In some cases, even dogs with normal thyroid function can have positive TgAA results, while dogs with clear clinical signs of hypothyroidism might show negative TgAA results. Therefore, the negative TgAA result in our case does not conclusively exclude immune-mediated hypothyroidism. In addition, TgAA testing, particularly in the later stages of the disease can have negative results.

In PAS, the presence of circulating autoantibodies is a common finding. The fundamental cause of the observed phenomenon can be attributed to a reduction in the activity of suppressor T cells (1). These cells, also known as regulatory T cells (Tregs), play a pivotal role in maintaining immune homeostasis by suppressing excessive immune responses and preventing autoimmunity. The decrease in Treg activity can lead to dysregulated immune responses, characterized by an inability to suppress pathogenic self-reactive T cells effectively. Tregs exert their suppressive function through various mechanisms, including the secretion of immunosuppressive cytokines such as IL-10, Transforming growth factor beta (TGF-β), and interleukin-35 (IL-35), consumption of interlukin-2 (IL-2), and induction of effector cell death via granzyme and perforin (20). The suppression of T cell responses by Tregs is crucial for preventing inflammatory diseases and maintaining tolerance to self-antigens. However, a decrease in Tregs function can result in the loss of this tolerance and the development of autoimmune conditions. Subsequently, the diminished activity of suppressor T cells is a significant factor in the perturbation of immune equilibrium, leading to heightened susceptibility to autoimmune diseases and uncontrolled immune reactions.

IL-10, a regulatory cytokine with potent anti-inflammatory and immunosuppressive properties, plays a critical role in maintaining immune homeostasis and preventing pathological immune responses. By suppressing pro-inflammatory cytokines and immune cell activity, IL-10 limits excessive inflammation and tissue damage, particularly in chronic and autoimmune conditions. Insufficient production or dysregulation of IL-10 disrupts the balance between pro-inflammatory and anti-inflammatory responses, contributing to the progression of autoimmune diseases such as rheumatoid arthritis, multiple sclerosis, and systemic lupus erythematosus. Experimental models demonstrate that IL-10 deficiency leads to exaggerated immune responses and severe tissue damage, while in humans, reduced IL-10 expression or function correlates with worsened disease outcomes (21).

There has been a study indicating that oclacitinib reduces both the production of IL-10 cytokine and the number of CD4+ and CD8+ T cells that produce this cytokine (22). Oclacitinib directly decreases IL-10 production by suppressing the activation and differentiation of IL-10-producing cells, including CD4+ T cells, CD8+ T cells, and Type 1 regulatory T (Tr1) cells, a specialized subset of CD4+ T cells with high IL-10 production. Moreover, oclacitinib indirectly inhibits IL-10 signaling by blocking the JAK-STAT pathway (23), which is essential for the immunosuppressive effects mediated by IL-10. Together, these mechanisms allow oclacitinib to exert a dual inhibitory effect on IL-10-mediated immune regulatory pathways, reducing both IL-10 levels and its downstream signaling.

Based on previous findings (22, 23), we hypothesized that oclacitinib-induced IL-10 suppression might contribute to PAS development as an environmentally triggered condition. In this study, IL-10 levels were measured in the affected dog and compared with those of 9 healthy controls. The affected dog's IL-10 concentration fell within the 95% confidence interval (CI) of IL-10 levels observed in healthy dogs, but this measurement was taken approximately 3 months after oclacitinib administration. Since prior studies observed IL-10 suppression only within 72 h of administration (22), it is likely that our measurement timing missed this transient suppression. These findings suggest that IL-10 suppression by oclacitinib may be transient and detectable only shortly after administration. However, prolonged use of oclacitinib might lead to chronic immune dysregulation, potentially creating a pro-inflammatory environment and contributing to autoimmune diseases like PAS. Previous study has reported that transient immune dysregulation can lead to long-term autoimmune responses (24). Transient depletion of Treg cells induced autoreactivity in CD4+ T cells, and despite the subsequent recovery of Treg cell levels, autoimmune responses persisted. This finding suggests that once immune homeostasis is disrupted, it may not automatically return to its original state. In the present case, oclacitinib-induced reductions in Treg cells and IL-10 may have contributed to the disruption of immune balance, potentially promoting the development of PAS. Furthermore, in this case, such immune dysregulation did not occur only once but rather repeatedly. Given that oclacitinib was administered continuously, the long-term cumulative effects of transient reductions in Treg cells and IL-10 likely contributed to progressive immune imbalance. In this case, these processes likely occurred multiple times, further increasing the likelihood of PAS development.

Additionally, although serum IL-10 level was within the normal range, once immune balance is disrupted, its restoration is not necessarily immediate. Specifically, systemic serum IL-10 concentrations do not always correlate with local IL-10 expression in target tissues, and IL-10 may have been insufficient in organs affected by autoimmune responses, such as the adrenal and thyroid glands (25). IL-10 plays an immunosuppressive role, but its expression is regulated in a tissue-specific manner and may be suppressed under certain conditions. The referenced study also emphasized that IL-10 expression varies among tissues and that the presence of systemic IL-10 does not guarantee uniform expression across all organs. For instance, in tissues where autoimmune responses have been triggered, the inflammatory microenvironment may suppress IL-10 expression, and IL-10 receptor sensitivity may also differ among tissues. Thus, even if serum IL-10 levels fall within the normal range, the availability of IL-10 in critical target organs for immune regulation may have been insufficient. This observation holds significant relevance in the present case. In PAS, the primary target organs—the adrenal and thyroid glands—may have experienced local IL-10 deficiency or impaired IL-10 signaling, potentially contributing to the persistence of autoimmune responses. Consequently, systemic IL-10 concentrations alone cannot adequately reflect immune regulatory capacity, and tissue-specific expression and function must be considered as crucial factors.

However, this study does not conclusively demonstrate IL-10 suppression, as only systemic serum IL-10 levels were measured, without assessing IL-10 expression in specific target organs. Further research, including large-scale studies measuring IL-10 levels at multiple time points and across specific tissues, is needed to clarify the role of IL-10 suppression in PAS pathogenesis.

This study specifically emphasizes a possible relationship between oclacitinib administration and the onset of PAS. Oclacitinib, while effectively managing itching and inflammation in dogs with allergic dermatitis through targeting the JAK1 pathway and inhibiting cytokines associated with inflammation, may also inadvertently alter the delicate balance of immune regulation. Such alteration can initiate autoimmune processes targeting multiple endocrine organs, thus potentially increasing the risk of infections and tumors, and raising the likelihood of developing autoimmune diseases like PAS (26). These findings highlight the complexity of oclacitinib's effects on immune homeostasis, underscoring the need for vigilant monitoring and careful management of endocrine function in dogs receiving this medication. While the therapeutic benefits of oclacitinib, such as rapid and sustained symptom relief, offer a relatively safe profile compared to other steroid medications, the long-term use of this drug requires that the benefits and risks be carefully weighed to tailor its use to individual patient needs. Routine monitoring may facilitate the early detection of PAS, enabling timely intervention and management of this complex interplay between therapeutic efficacy and potential adverse effects.

In conclusion, this case underscores the necessity of sustained research to unravel the long-term implications of oclacitinib on immune homeostasis and its potential link to autoimmune disease development.

## Conclusion

This case suggests a potential association between prolonged oclacitinib administration and the development of PAS in a dog. Regular hormonal monitoring and careful dose adjustments of oclacitinib during long-term therapy of atopic dermatitis are recommended to minimize the risk of autoimmune disease development.

## Data Availability

The original contributions presented in the study are included in the article/supplementary material, further inquiries can be directed to the corresponding author.
